# Assessing the Online Social Environment for Surveillance of Obesity Prevalence

**DOI:** 10.1371/journal.pone.0061373

**Published:** 2013-04-24

**Authors:** Rumi Chunara, Lindsay Bouton, John W. Ayers, John S. Brownstein

**Affiliations:** 1 Department of Pediatrics, Harvard Medical School, Boston, Massachusetts, United States of America; 2 Children’s Hospital Informatics Program, Division of Emergency Medicine, Boston Children’s Hospital, Boston, Massachusetts, United States of America; 3 Harvard School of Public Health, Boston, Massachusetts, United States of America; 4 Graduate School of Public Health, San Diego State University, San Diego, California, United States of America; University of Catania, Italy

## Abstract

**Background:**

Understanding the social environmental around obesity has been limited by available data. One promising approach used to bridge similar gaps elsewhere is to use passively generated digital data.

**Purpose:**

This article explores the relationship between online social environment via web-based social networks and population obesity prevalence.

**Methods:**

We performed a cross-sectional study using linear regression and cross validation to measure the relationship and predictive performance of user interests on the online social network Facebook to obesity prevalence in metros across the United States of America (USA) and neighborhoods within New York City (NYC). The outcomes, proportion of obese and/or overweight population in USA metros and NYC neighborhoods, were obtained via the Centers for Disease Control and Prevention Behavioral Risk Factor Surveillance and NYC EpiQuery systems. Predictors were geographically specific proportion of users with activity-related and sedentary-related interests on Facebook.

**Results:**

Higher proportion of the population with activity-related interests on Facebook was associated with a significant 12.0% (95% Confidence Interval (CI) 11.9 to 12.1) lower predicted prevalence of obese and/or overweight people across USA metros and 7.2% (95% CI: 6.8 to 7.7) across NYC neighborhoods. Conversely, greater proportion of the population with interest in television was associated with higher prevalence of obese and/or overweight people of 3.9% (95% CI: 3.7 to 4.0) (USA) and 27.5% (95% CI: 27.1 to 27.9, significant) (NYC). For activity-interests and national obesity outcomes, the average root mean square prediction error from 10-fold cross validation was comparable to the average root mean square error of a model developed using the entire data set.

**Conclusions:**

Activity-related interests across the USA and sedentary-related interests across NYC were significantly associated with obesity prevalence. Further research is needed to understand how the online social environment relates to health outcomes and how it can be used to identify or target interventions.

## Introduction

The rapid increase in the population prevalence of obesity worldwide suggests the important role of environmental effects [1]–[3]. Previous studies have developed metrics to explore the relationship between the built environment and obesity with mixed results [4], [5]. For example, higher levels of physical activity and lower population obesity prevalence were found in high- versus low-walkability neighborhoods [4] and prevalence of overweight children was found to be correlated with hours of television viewed [6]. However, the so-called poor urban “food desert’s” effect on obesity has been called into question [7]. These assorted results motivate study of the impact of other environmental factors in relation to obesity. Beyond the built environment, there is evidence for the social environment’s influence on obesity in both individual [8], [9] and community settings [10]–[13]. This has been hypothesized by multiple rationales: socially connected individuals share similar experiences, events, influences, and support which lead to simultaneous weight gain or loss, people choose to associate with others like them (homophily), and individuals exert influence on others [8], [10], [12], [14].

Despite these initial findings, research on the relationship between social environment and health outcomes lags behind its built environment counterpart due to a lack of robust tools for studying the social environment. Metrics of the social environment have been elucidated via relations (for example friendships or family members), through pre-collected data from existing longitudinal studies [8], interviews and surveys [12], or wireless sensors [15]. These methods are limited in scope by their cost, required labor, maximum size and granularity of population they can be used to examine, and their reliance on user participation and recall. This has limited study of the relationship between the social environment and health outcomes to particular groups and individual-level studies [10], [12], [13].

Social media offers a new opportunity for learning about social environments. Previous studies have shown certain data from social media platforms to be a good corollary for public health events [16]–[19]. An open question is how the online social environment is related to real-world health outcomes, such as the population prevalence of obesity. Recently an online social network was harnessed to explore the role of messaging on a real-world behavior (voting) via a substantial 61-million-person cohort. This pioneering study highlighted the potential of online social networks as a medium for understanding a component of the social environment of populations, the online social environment [20].

Accordingly, we used the largest online social network in the United States (and the world) to better understand the social environment’s potential association with population obesity. Herein, the online social environment’s relationship to the prevalence of obese and/or overweight people in metropolitan and neighborhood-scale populations was investigated. It was hypothesized that greater online interests in activity will be associated with lower prevalence of obese and/or overweight people while greater online interests around television will be associated with a higher prevalence of obese and/or overweight people.

## Methods

### Data Sources and Selection

#### Facebook

Online social networks offer a new form of observational data that describe the social environment. These networks engage millions of unique users monthly [21]. Facebook is an online social network where individuals can dynamically enter information on their background, demographics and interests. Approximately one-half of the United States and one-eighth of the entire world population is active on Facebook as of June, 2012 [22]. The Facebook Advertisement platform allows advertisers to target users by characteristics including age, location and interests as determined by their profile information. The platform provides the number (found to be updated approximately weekly) of users who fall under the selected categories and demographics at the resolution of zip code, city, state, or country including surroundings at varying geographic radii. Categories are determined through individuals’ wall postings, likes and interests that they share with their Facebook friends and through which they create a social milieu.

In order to evaluate our hypothesis, we examined activity and sedentary related interest categories from Facebook that have, in other traditional studies of the social environment, been positively or negatively related to obesity [23]. We found that that population prevalence of a seemingly activity-related interest in “sports” was negatively correlated with population prevalence of other activity-related interests; “sports” may indicate an interest in watching sports instead of relating to an individual’s health per se. Accordingly we selected two clearly activity-related interest categories: “health and wellness” and “outdoor fitness activities”, and used the proportion of users with interest in both of these for increased specificity. Conversely, because sedentary behaviors, particularly television watching, have been shown to be negatively associated with obesity [24], [25], this category was selected to examine association between sedentary-related interests in the social environment and obesity.

The proportion of users with each interest by city for the USA (or by zip code for NYC) was obtained from Facebook and the values for the major cities in each SMART (defined below) metropolitan or micropolitan area (USA) and zip codes in each neighborhood (NYC) was summed. We accounted for different levels of Facebook activity by normalizing the number of users in a particular location with interest in “health and wellness” as well as “outdoor fitness activities” (or “television”) by the number of users in that location with any of the interest categories. Totals were restricted to users aged 18–64 (inclusive). The interest data was collected over the course of one week starting April 14 (USA activity-related data), and May 11 (NYC). The “television” interest data for USA was collected on May 17, 2012. In total the number of Facebook users considered across the nation was 57 339 270, and within NYC was 8 206 240.

#### Behavioral Risk Factor Surveillance System and the Selected Metropolitan/Micropolitan Area Risk Trends (SMART) Project

We chose the Centers for Disease Control and Prevention’s Behavioral Risk Factor Surveillance System (BRFSS) for our USA outcome measure: prevalence of obese and/or overweight people. The BRFSS is a cross-sectional telephone survey conducted by the Centers for Disease Control and Prevention (Office of Surveillance, Epidemiology, and Laboratory Services) and state health departments in the United States. The BRFSS was designed to produce state-level estimates, however growth in the number of respondents has made it possible to produce prevalence estimates for smaller areas and led to the Selected Metropolitan/Micropolitan Area Risk Trends (SMART) project [26]. This project offers health officials access to local-level data with the objective of helping local health officials plan, implement, and evaluate their prevention efforts, identify emerging health problems, establish and track health objectives, and develop and evaluate public health policies and programs. For each of the years considered here (2010, 2007, 2005 and 2003), SMART provides data for 189 metropolitan and micropolitan statistical areas which each had at least 500 completed interviews [27].

#### New York City EpiQuery Community Health Survey

In order to evaluate an alternate geographic level for the relationship between the social environment and prevalence of obese and/or overweight people, data from Facebook and the New York City Community Health Survey (CHS) was used. The CHS is an annual telephone survey by New York City Department of Health providing data on the health of New Yorkers. The survey provides neighborhood, borough and citywide resolution of data on chronic diseases and behavioral risk factors [28]. The CHS data includes 34 neighborhoods in New York City, with a total completed survey sample size of 8 665 in 2010 [28].

For our analysis an outcome variable was chosen from the BRFSS and CHS surveys that represents a measurable health outcome, opposed to health behaviors or descriptions which are more subjective and subject to biases. The BRFSS and CHS surveys both include questions on height and weight, which are used to calculate the Body Mass Index (BMI), a simple index that is commonly used to classify overweight and obesity in adults and is deemed to be a measure of high reliability and high validity [29], [30]. The World Health Organization definition provides that someone who is obese has as a BMI greater than or equal to 30, and someone who is overweight has a BMI greater than or equal to 25 [31]. The published data sets both specifically describe the proportions of the population who are overweight and who are obese (those classified as obese are also overweight and here we refer to a population with a BMI greater than or equal to 25 as those who are “obese and/or overweight”). At the time of writing, the most recent data from both the BRFSS SMART project and the CHS was from 2010. BRFSS and CHS data from previous years (2007, 2005 and 2003) was also analyzed to understand how associations might fluctuate over time.

### Analysis

To evaluate the relationship between our predictor and outcome variables, and in order to account for both sampling error and fundamental uncertainty, we used a Monte Carlo simulation approach for the linear regression [32]. Using the Facebook population interest prevalences as predictors and outcomes from BRFSS and CHS, we first obtain the estimated regression coefficients for a simple linear regression, and variance-covariance matrix for each relation. Then, we simulated the parameters from a multivariate normal distribution, using a built-in program [33]. 1000 randomly drawn estimates were made from a sampling distribution with mean equal to the maximum likelihood point estimates of the varying components of the predictors and variance equal to the variance covariance matrix of the estimates. This returned the expected value of the outcomes (BRFSS or CHS proportions of obese or overweight people), which were averaged to yield one estimate of the expected value. Sorting these probabilities and recording the values in the 25th and 976th percentile positions generated a 95 percent confidence interval, illustrating confidence in the breadth and relationship of the entire set of data.

To evaluate the predictive performance of the data across the country (for the significant associations), we used *k*-fold cross validation. This method intuitively shows how a subset of Facebook and BRFSS data from the country could be used to predict health outcomes in remaining metropolitans, given Facebook data only. Specifically, we split the complete data set into 10 (*k*) mutually exclusive subsets of approximately equal size with the model being trained *k* times and compared to the test data [34].

## Results

**Figure 1:**
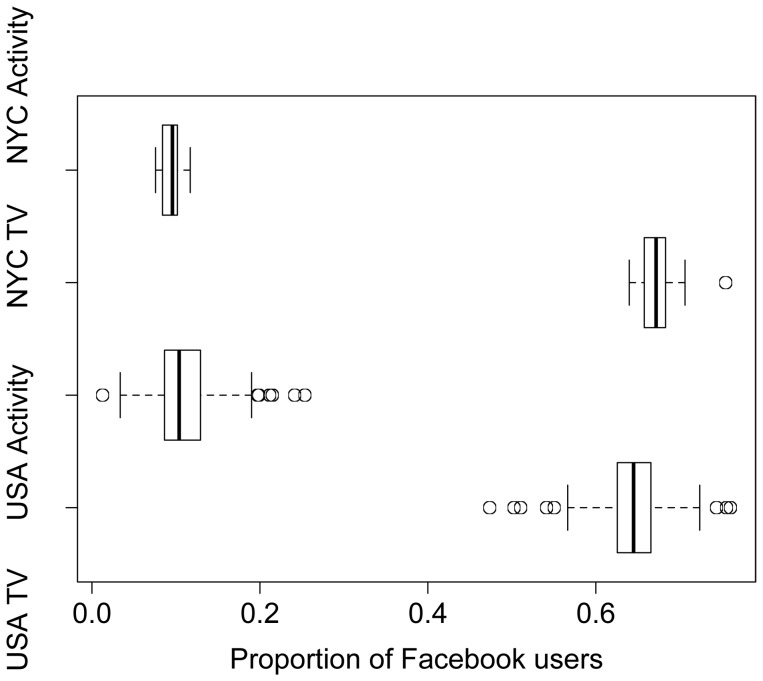
Range of proportions of users by interest category and geography. Proportion of Facebook users across neighborhoods in NYC and metropolitans or micropolitans in USA with activity-related interests or interest in television.

The number of Facebook users who had interest in the activity categories by metropolitan or micropolitan ranged from 140 (Wauchula, FL) to 127 700 (Los Angeles-Long Beach-Glendale, CA). Total number of users in these cities ranged from 4 720 to 2 951 260. Figure 1 shows the range of proportion of Facebook users who have interests in activity or television categories, nationally and within NYC. Nationally, proportion of users with activity-related interests by metropolitan ranged from 0.013 (Kansas City, MO-KS) to 0.254 (Coeur d’Alene, ID), while those with interest in television ranged from 0.503 (Eugene-Springfield, OR) to 0.760 (Myrtle Beach-Conway-North Myrtle Beach, SC). In NYC, proportion of users with activity-related interests ranged from 0.076 (Southwest Queens) to 0.112 (Coney Island) and interest in television from 0.640 (Greenpoint) to 0.706 (Northeast Bronx). The activity-related and television-related interest ranges overlap (Figure 1), demonstrating consistency between proportions across the entire nation and within one city. Overall, there was more television interest than activity-related interests at both the metropolitan and micropolitan level across the USA and neighborhood level within NYC.

**Figure 2:**
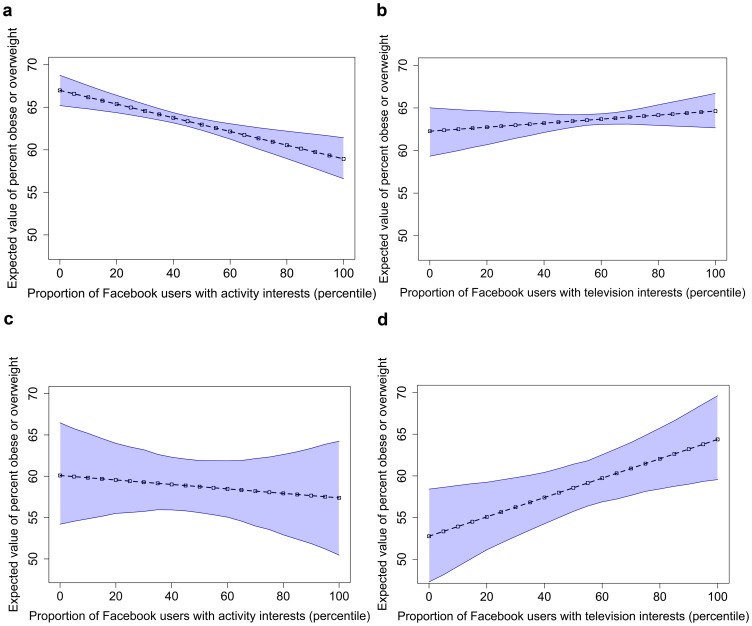
Relationship between interests and obesity prevalence. Greater proportion of users with activity related interests (a [USA] and c [NYC]) corresponds to lower prevalence of obesity. Greater proportion of users with television interests corresponds to higher prevalence of obesity (b [USA] and d [NYC]). Dashed lines are the mean expected value of proportion of overweight or obese in the population and points show the exact values. 95% confidence intervals illustrated via the shaded region, which outlines the dashed line.

**Figure 3:**
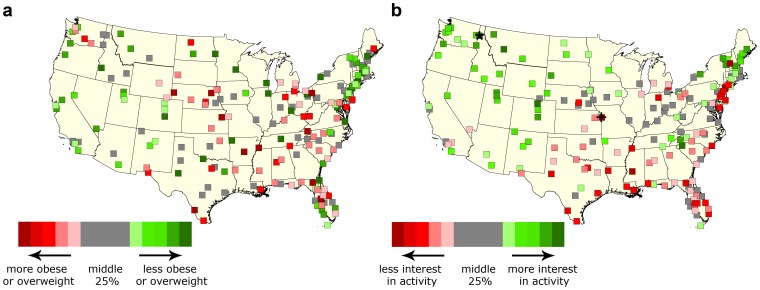
Prevalence of activity-related interests and obesity in the USA. Squares for each metropolitan or micropolitan used in the study, color-coded by (a) population prevalence of obese and/or overweight people or (b) the proportion of the population with activity-related interests. Metropolitans in grey are in the middle 25% based on proportion of individuals. Proportion of obese or overweight is color-coded from red to green (more to less) (a), and proportion of activity-related interests from red to green (less to more) (b). For the data from Facebook (b), the place with the minimum proportion of people with activity-related interests is demarcated by “+”, and place with maximum proportion by “*”.

**Figure 4:**
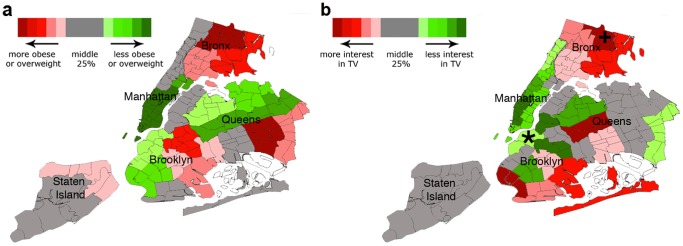
Prevalence of television interests and obesity in New York City. Neighborhoods in NYC are color-coded based on the (a) population prevalence of obese and/or overweight people or (b) the proportion of the population with television-related interests. Neighborhoods in grey are in the middle 25% based on proportion of individuals. Proportion of obese or overweight is color-coded from red to green (more to less) (a), and proportion of television-related interests from red to green (more to less) (b). For the data from Facebook (b), the neighborhood with the minimum proportion of people with activity-related interests is demarcated by “+”, and neighborhood with maximum proportion by “*”.

From the linear regression, an increase from the minimum to maximum proportion of users with activity-related interests online across the country (values above) was associated with a significant 12.0% (95% CI 11.9 to 12.1, p<0.0001) lower prevalence of obese and/or overweight people in metropolitans or micropolitans (USA, 2010). In 2010 NYC, this resulted in a 7.2% (95% CI: 6.8 to 7.7, p = 0.44, not significant) lower prevalence of obese and/or overweight people in all neighborhoods. An increase in proportion of users with online interest in television was associated with a higher prevalence of obese and/or overweight people of 3.9% (95% CI: 3.7 to 4.0, p = 0.32, not significant) (USA, 2010) and a significant increase of 27.5% (95% CI: 27.1 to 27.9, p<0.005) (NYC, 2010) (Figure 2). Geographically, the distributions of metropolitans across the USA and NYC with high prevalence of obese and/or overweight people are reflected in the distributions of metropolitans with relatively lower activity interests and higher television related interests, respectively (Figures 3 and 4).

The range of mean square prediction errors for the 10 folds of the cross validation, each using 90% of the national Facebook activity-related data, was 2.84 to 5.16. Thus the average root mean square prediction error was 4.23, which is comparable to the root mean square error of a model using the entire dataset, 4.24. The similarity between the mean square prediction error and the mean square error of the model indicates that the model subsets demonstrate good predictive ability [35].

Evaluating the Facebook data against historical prevalences of obese and/or overweight people from BRFSS and CHS (2007, 2005 and 2003) showed the same trends as for the 2010 data (all comparisons significant with p<0.05 except NYC 2007 and 2003, Table S1).

## Discussion

This study investigated the relationship between the online social environment, across cities in the USA and neighborhoods within NYC, with a real-world health outcome: the population prevalence of obese and/or overweight people. As hypothesized, higher activity-related interests online were associated with a lower prevalence of obese and/or overweight people. And conversely, higher interest in television online was associated with a higher prevalence of obese and/or overweight people. The associations between prevalence of obese and/or overweight people and online interest in television in the USA and online activity-related interests in NYC were not significant, however both trended in the expected directions. The levels of both activity- and inactivity-related interests were similar at the metropolitan and micropolitan level in the United States, and the neighborhood level in New York City, suggesting the association of the social environment and population prevalence of obesity at multiple geographic levels. We also found that the proportion of individuals interested in activity and television within one city (NYC) was more similar than across the nation. This work suggests the potential of the online social environment to be used in surveillance and prediction of obesity prevalence in populations across cities and neighborhoods.

### Strengths and Weaknesses of the Study

A main strength of this study is that we demonstrated a new method for understanding the relationship between an aspect of the social environment and a real world health outcome. As well, we were able to consider an extremely large population of people, many times larger than populations in traditional cohorts or studies such as in the BRFSS and CHS data used here [22]. Although the time points for our data sources did not overlap (Facebook data was from mid-2012 and survey data was measured in 2010), we determined that comparison was justified because we found consistent associations between the Facebook data used here and historical BRFSS and CHS data for 7 years prior.

A primary limitation of any ecological study such as this is that inferences are not made at the individual level, and should not be deduced from those groups to which the individuals belong. The implication of this limitation in the work here is that the aggregate relationship of the social environment to health outcomes in specific cities or neighborhoods may differ from extreme situations for particular individuals. Another limitation of the study is that no causal relationships are proven. As well, since we used a cross-section of data from Facebook, the data only represents the status of the population at that one time period, which could be influenced by other temporal parameters that may or may not be related to our outcome (although we evaluated the relationship to historical data as discussed above). A direct comparison of the predicted ranges of obesity prevalence to ranges in the BRFSS and EpiQuery data sets is difficult due to outliers. However, it is clear that the predicted ranges do not extend the full range of the BRFSS and EpiQuery data (Figure S1), indicating that addition of other factors into the model are needed to elucidate more of the variation. Regarding the information used, as with any data source, there are limitations and biases in using online social networks to understand the social environment. The population of Facebook users is limited to those who have access to the Internet and further, to those who choose to engage with the platform. However, this population is growing rapidly worldwide [36]. Another limitation of our study is the lack of detailed information about the social networks of the individuals in each geographic area; the influence of the social and built environments cannot be precisely disentangled through this study (for example, places with environments more conducive to activity may include or select for people with more activity-related interests and less prevalence of obesity). Also, we do not use information about specific network connections, which studies examining the social environment and obesity on an individual level have done [8]. For this study, although the BRFSS and CHS data are demographically weighted and age-adjusted to represent the population [28], [37], the Facebook data used is not weighted. This could be a source of misrepresentation. In terms of age, younger people, who are more numerous on Facebook, could have different levels of interest in each of the categories. However, it was found that after normalization by the number of people with any online interests, the trends across cities of activity-related interests by age group were similar (Figures S2 and S3). Finally, from the Facebook Advertisement platform, there are no metrics provided about precision of estimates, however the information is freely available, and resolution was accurate enough for the purposes of this study. A limitation of our outcome data is that the cut-off for obese or overweight BMI has been found to vary by race [38], and consequently results of this study may have potential biases in neighborhoods or cities with more Asians.

### Strengths and Weaknesses in Relation to Other Studies

The findings in this study add to a growing body of research highlighting the important clinical and public health implications of the social environment [8], [10], [12], especially in regards to obesity. While previous studies of the social environment and health are focused on particular populations or person-to-person spread, our findings relate the social environment at the level of neighborhoods and metropolitans (for the entire 18 to 64 population). As well, other studies have generally used surveying to uncover aspects of the social environment including social capital and social norms, but have not examined the online social environment specifically, which is a growing component of the overall social environment. This study corroborates the association of social environments and obesity, and also begins to uncover aspects of the environment, such as interests in the online medium, and how they are positively or negatively related to this outcome. Sharing of these norms through Facebook may also be magnified because network connections are “friends”; people who likely share demographic profiles, meaning there messages are better focused [39]. In comparison to other studies using data from Facebook and not performed in conjunction with Facebook, this study considers a relatively large population opposed to select groups [40], [41]. To our knowledge, our study is the first to relate the online social environment to real world health outcomes in populations. On the other hand, because this study is ecological, we are not able to relate our results to the actions or effects of individuals, which have been done before in studies of social environment and obesity and in studies of large populations using Facebook [8], [42].

### Meaning of the Study: Possible Mechanisms and Implications for Clinicians or Policymakers

Here we present online social networks as a new mechanism for public health surveillance of real-world health outcomes. Accessibility of online data also presents the opportunity to use data-mining approaches to understand how aspects of the online environment correspond best to different health outcomes. Conversely, because results here suggest the possibility that both positive and negative health outcomes (higher or lower prevalence of obesity) can be related to online social network interests, the online social environment could be harnessed for intelligently targeted health interventions, such as through online and mobile messaging. While activity-related interests in the USA and sedentary-related interests in NYC showed a significant association with the obesity outcome, in the case of weak discriminatory effects this platform can also be used in conjunction with other intervention methods or programs. Although we used just a cross-section of the online interest data in this study, a benefit of online social networks is that they can provide data in real-time, while traditional measures of the social environment are limited by low measurement frequency. Specifically, the most recent data available for the outcome measures was for over a year prior to the online environment data, and only in yearly time points. As well, data from online social networks is available passively, can be obtained unobtrusively and are not subject to survey and recall bias and limited worldwide reach of other population-level data collection methods that have been used in research and surveillance of obesity [8].

Further, in contrast to other online social mediums such as Internet search queries, online social networks also present a unique opportunity to obtain real-time user data along with detailed interaction information. The online social network medium can also be used to understand the psychosocial interactions that could influence health outcomes, such as feelings of acceptability, behavior of friends and contacts or knowledge of services and support. The large user base of these networks enables a substantial or select population from around the world to be reached at a variety of levels. This can facilitate understanding of the different geographic levels at which sources of influence may arise, and inform implementation of location-specific interventions. Additionally, high user interaction and measurement indicate that online social networks could be used to rapidly gauge responses to traditional interventions or rapidly changing factors such as a city or community-wide health initiatives [43], [44] without any temporal delays or gaps, due to the continuous nature of the information. This would augment data collected through health institutions and official reporting structures for which acquisition requires more time and labor. The online social environment is becoming more relevant for populations worldwide [22], [36] and this modality offers a real-time, ease of access, low-cost population-based approach to public health surveillance, beyond an individualized clinical picture.

### Future Research

Through information from the online social network Facebook, it was shown that greater television related interests, or less physical activity related interests online for populations in metropolitans and micropolitans across the USA or neighborhoods across NYC were associated with a higher population prevalence of obesity. As well, we showed that activity-related online interests in the USA could be predictive of population obesity and/or overweight prevalence. To grow on the utility of the online social environment in public health surveillance demonstrated here, further research can use a more data-driven approach to understand more aspects of the online social environment that are related to real-world health outcomes. Additionally, future work can harness the availability of real-time data from this source in order to decrease obesity prevalence by targeting and monitoring health interventions through online networks in parallel with traditional public health measures, in a longitudinal manner. Time-series of data from online social networks could also be used to investigate causality between the online data and obesity prevalence. The online social environment can also be used to investigate how the social environment results in different outcomes for different populations, which in turn would help identify characteristics of populations that may lead to health outcome disparities between communities. Obesity is one of many significant health outcomes worldwide and the relationship between the online social environment and health outcomes should be evaluated further for other global health issues.

## Supporting Information

Figure S1 Range of obesity prevalence levels, USA and NYC. The prevalence of obese and/or overweight people in metropolitans or micropolitans in the USA, sequentially (a) and in the neighborhoods in NYC (b) used in this study.
(TIF)

Figure S2 Interest levels in cities weighted by age groups. Interest in ‘Outdoor Fitness Activities’, one of the components of our Facebook Health Metric, for a select group of cities by age group (a) normalized to the number of people in that age group on Facebook and (b) normalized to the number of people in that age group on Facebook with any interests. Once the data was normalized to the number of people in each age group who have any interests, the trends between age groups across cities become more consistent. However, the overall trends city-to-city are similar in the case where the data is only normalized by the number of people overall.
(TIF)

Figure S3 Correlation between interest levels in cities by age groups. Correlation of interests by age group, across each of the cities considered in Figure S2. While correlations between age groups across cities range from (0.08 to 0.91) when only taking into account the total number of people in each age group (a), once the number of people in each age group with interest in OFA are normalized to the total in their age group with any interests (b), correlation across cities are all highly increased (range from 0.84 to 0.99).
(TIF)

Table S1 Lagged comparison between Facebook data and health outcome information. Significance of association between Facebook interest data (Interests) obtained in April–May 2012 and health outcomes for USA and NYC (Geography) in 2003, 2005 and 2007. All of the associations between activity-related interests and historical obesity data for the USA are significant (p<0.05). Associations between television-related interests in NYC are significant in 2005, and all comparisons trend in the expected directions (higher prevalence of obese and/or overweight people for lower proportion of population with activity-related or higher proportion of population with TV-related interests).
(DOCX)
