# Supplementary figures and images for: Assessing the Online Social Environment for Surveillance of Obesity Prevalence
Source: PLoS One. 2013 Apr 24;8(4):e61373. doi: 10.1371/journal.pone.0061373 (PMC3634787; doi:10.1371/journal.pone.0061373)

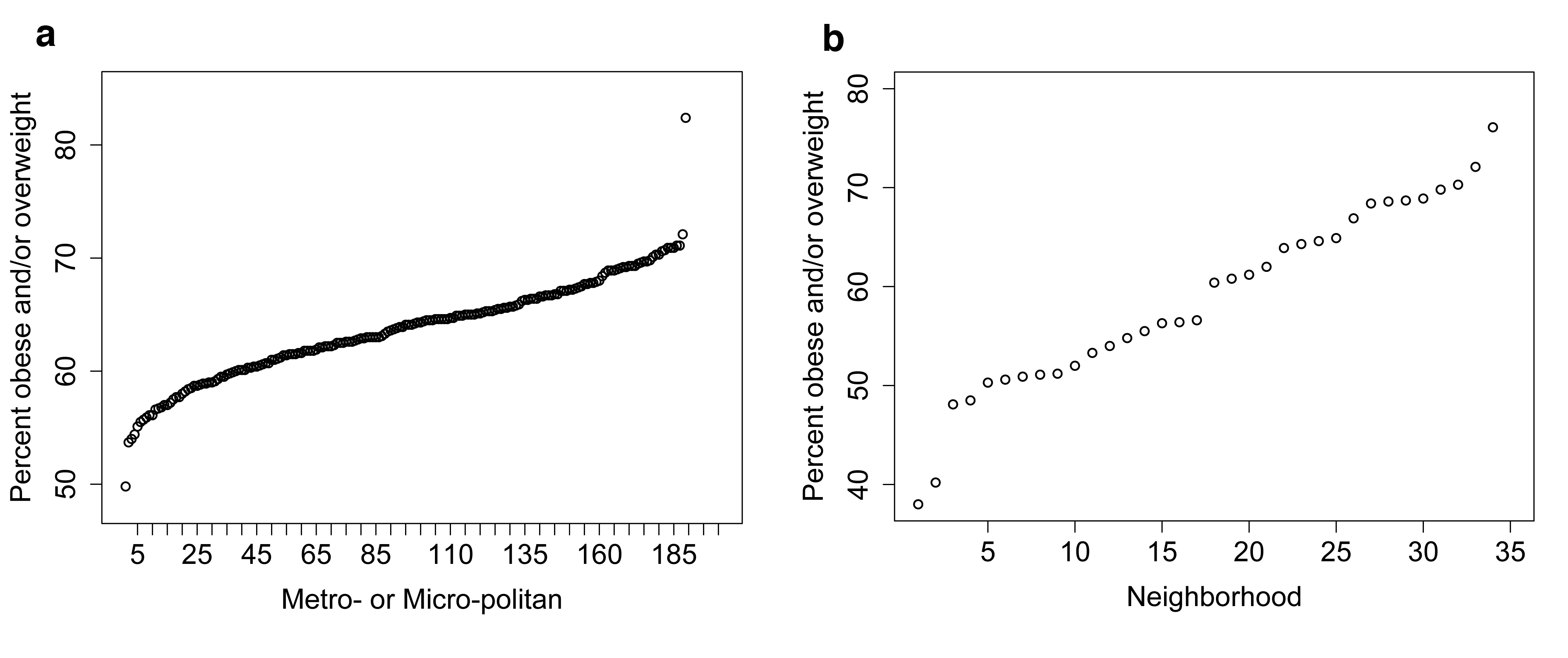

Supplement: Figure S1 — Range of obesity prevalence levels, USA and NYC. The prevalence of obese and/or overweight people in metropolitans or micropolitans in the USA, sequentially (a) and in the neighborhoods in NYC (b) used in this study. (TIF) [file pone.0061373.s001.tif]

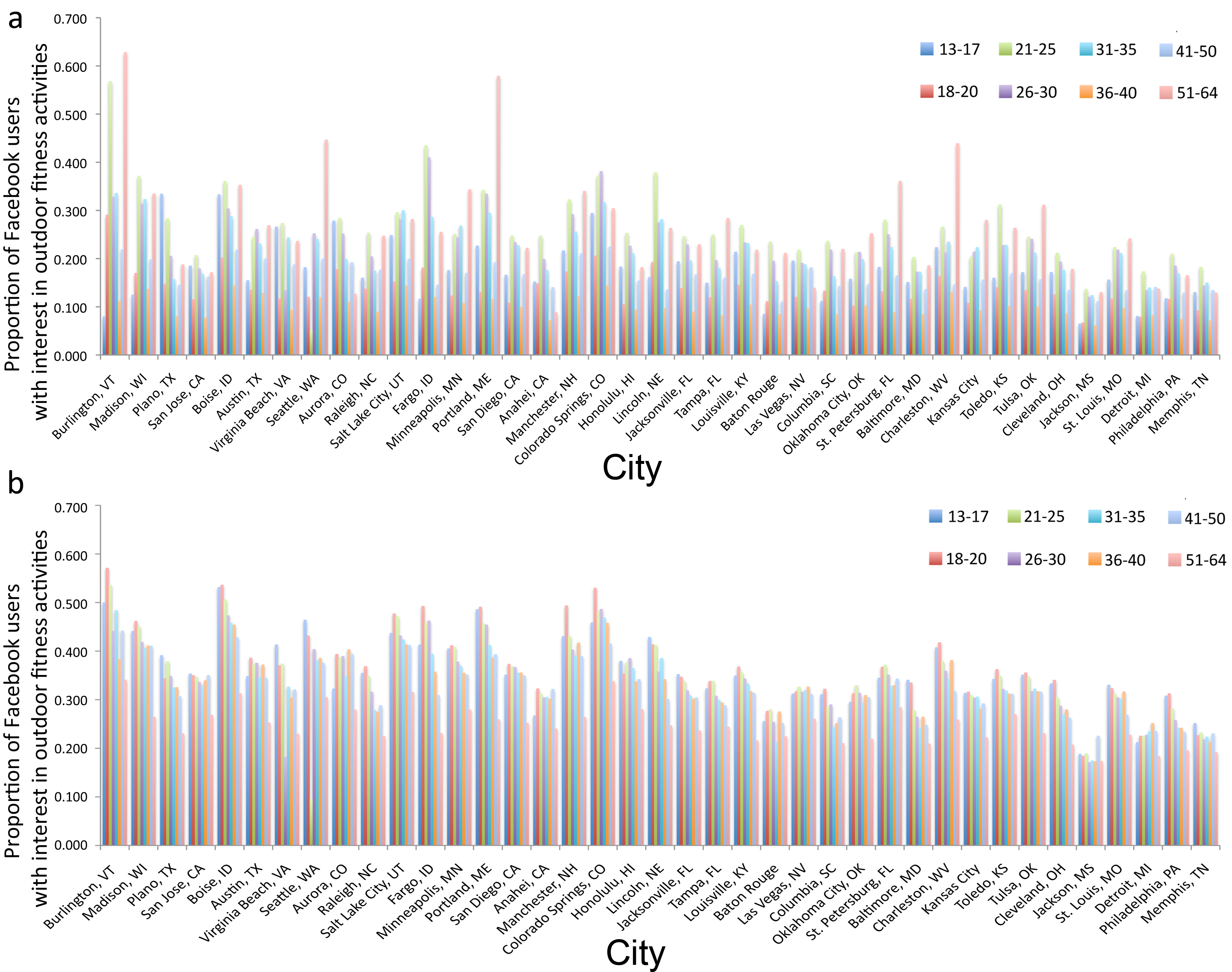

Supplement: Figure S2 — Interest levels in cities weighted by age groups. Interest in ‘Outdoor Fitness Activities’, one of the components of our Facebook Health Metric, for a select group of cities by age group (a) normalized to the number of people in that age group on Facebook and (b) normalized to the number of people in that age group on Facebook with any interests. Once the data was normalized to the number of people in each age group who have any interests, the trends between age groups across cities become more consistent. However, the overall trends city-to-city are similar in the case where the data is only normalized by the number of people overall. (TIF) [file pone.0061373.s002.tif]

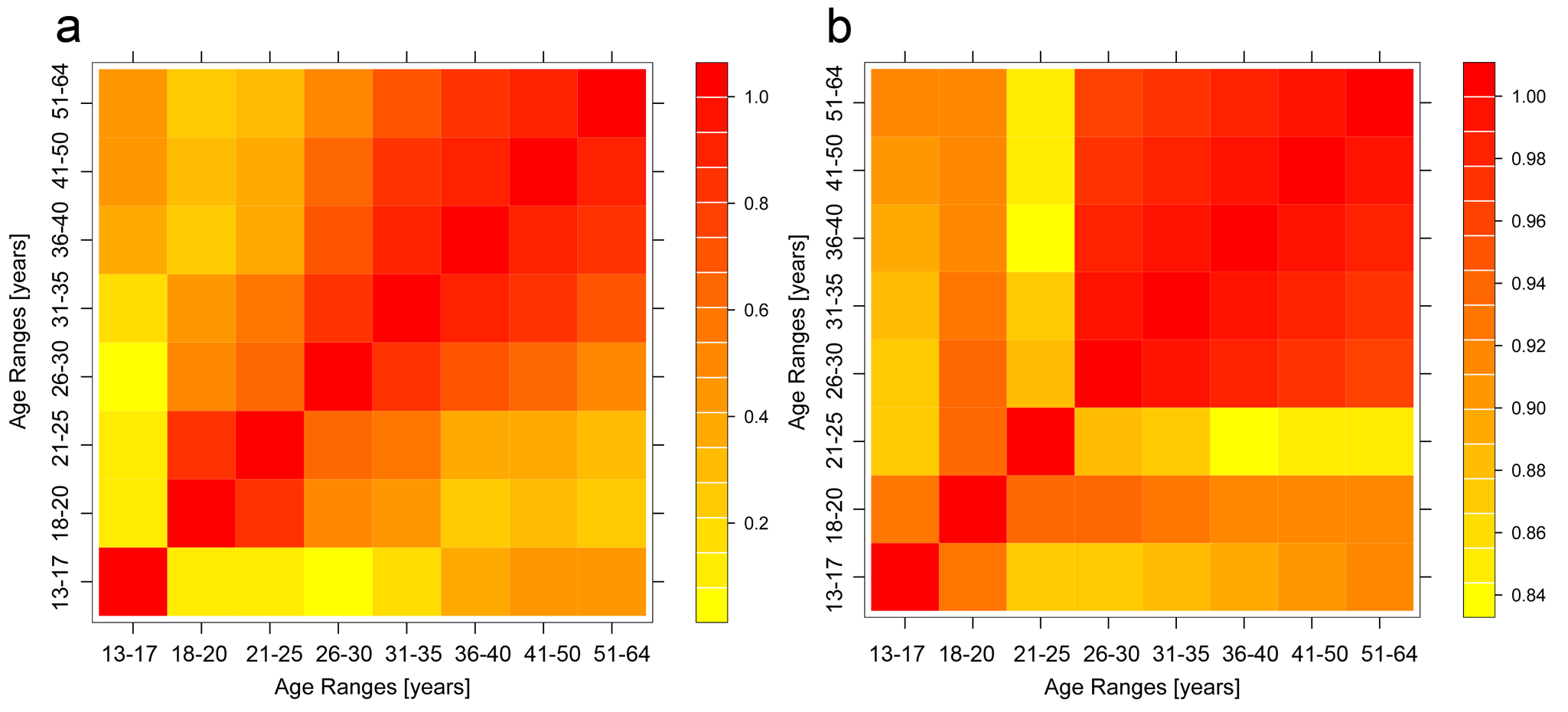

Supplement: Figure S3 — Correlation between interest levels in cities by age groups. Correlation of interests by age group, across each of the cities considered in Figure S2. While correlations between age groups across cities range from (0.08 to 0.91) when only taking into account the total number of people in each age group (a), once the number of people in each age group with interest in OFA are normalized to the total in their age group with any interests (b), correlation across cities are all highly increased (range from 0.84 to 0.99). (TIF) [file pone.0061373.s003.tif]
