# Supplementary material for: Assessing the Online Social Environment for Surveillance of Obesity Prevalence
Source: PLoS One. 2013 Apr 24;8(4):e61373. doi: 10.1371/journal.pone.0061373 (PMC3634787; doi:10.1371/journal.pone.0061373)
Supplement: Table S1 — Lagged comparison between Facebook data and health outcome information. Significance of association between Facebook interest data (Interests) obtained in April–May 2012 and health outcomes for USA and NYC (Geography) in 2003, 2005 and 2007. All of the associations between activity-related interests and historical obesity data for the USA are significant (p<0.05). Associations between television-related interests in NYC are significant in 2005, and all comparisons trend in the expected directions (higher prevalence of obese and/or overweight people for lower proportion of population with activity-related or higher proportion of population with TV-related interests). (DOCX) [file pone.0061373.s004.docx]

| **Year** | **Geography** | **Interests** | **p-value** | **Predicted trend** |
| --- | --- | --- | --- | --- |
| 2007 | USA | Activity | <0.0001 | -12.7% |
| 2005 | USA | Activity | 0.021 | -7.5% |
| 2003 | USA | Activity | 0.046 | -7.1% |
| 2007 | NYC | TV | 0.056 | +10.8% |
| 2005 | NYC | TV | 0.004 | +17.1% |
| 2003 | NYC | TV | 0.061 | +10.2% |
